# Integrative Genomic–Epigenomic Analysis of Clozapine-Treated Patients with Refractory Psychosis

**DOI:** 10.3390/ph14020118

**Published:** 2021-02-04

**Authors:** Yerye Gibrán Mayén-Lobo, José Jaime Martínez-Magaña, Blanca Estela Pérez-Aldana, Alberto Ortega-Vázquez, Alma Delia Genis-Mendoza, David José Dávila-Ortiz de Montellano, Ernesto Soto-Reyes, Humberto Nicolini, Marisol López-López, Nancy Monroy-Jaramillo

**Affiliations:** 1 Department of Biological Systems, Metropolitan Autonomous University-Xochimilco, Mexico City 04960, Mexico; yeryegibran@gmail.com (Y.G.M.-L.); blankita0807@gmail.com (B.E.P.-A.); betoov@yahoo.com.mx (A.O.-V.); mlopez@correo.xoc.uam.mx (M.L.-L.); 2 Department of Genetics, National Institute of Neurology and Neurosurgery, “Manuel Velasco Suárez”, Mexico City 14269, Mexico; djdodem@gmail.com; 3 Genomics of Psychiatric and Neurodegenerative Diseases Laboratory, Instituto Nacional de Medicina Genómica, SSA, Mexico City 14610, Mexico; jimy.10.06@gmail.com (J.J.M.-M.); adgenis@inmegen.gob.mx (A.D.G.-M.); hnicolini@inmegen.gob.mx (H.N.); 4 Natural Sciences Department, Universidad Autónoma Metropolitana-Cuajimalpa, Mexico City 05348, Mexico; esotoreyes@cua.uam.mx; 5 Grupo de Estudios Médicos y Familiares Carracci, Mexico City 03740, Mexico

**Keywords:** clozapine, mood stabilizer, refractory psychosis, pharmacogenomics, predictive model, methylome, polygenic risk scores

## Abstract

Clozapine (CLZ) is the only antipsychotic drug that has been proven to be effective in patients with refractory psychosis, but it has also been proposed as an effective mood stabilizer; however, the complex mechanisms of action of CLZ are not yet fully known. To find predictors of CLZ-associated phenotypes (i.e., the metabolic ratio, dosage, and response), we explore the genomic and epigenomic characteristics of 44 patients with refractory psychosis who receive CLZ treatment based on the integration of polygenic risk score (PRS) analyses in simultaneous methylome profiles. Surprisingly, the PRS for bipolar disorder (BD-PRS) was associated with the CLZ metabolic ratio (pseudo-R^2^ = 0.2080, adjusted *p*-value = 0.0189). To better explain our findings in a biological context, we assess the protein–protein interactions between gene products with high impact variants in the top enriched pathways and those exhibiting differentially methylated sites. The GABAergic synapse pathway was found to be enriched in BD-PRS and was associated with the CLZ metabolic ratio. Such interplay supports the use of CLZ as a mood stabilizer and not just as an antipsychotic. Future studies with larger sample sizes should be pursued to confirm the findings of this study.

## 1. Introduction

Antipsychotic drugs are effective in treating symptoms of psychosis and preventing relapses [1,2,3]. Psychotic symptoms (hallucinations, delusions, and distorted behavior) can be observed in different psychiatric disorders, such as schizophrenia (SZ), schizoaffective disorder (SD), bipolar disorder (BD), and even in major depressive disorder (MDD) [1,2,3,4,5,6]. Among these patients, about 30% are considered refractory, and clozapine (CLZ), an atypical antipsychotic, remains the treatment of choice for the population who has failed to improve on two other previous antipsychotic treatments [7,8,9]. CLZ has also been proposed as an effective mood stabilizer, although its mechanism of action is still unclear [10]. 

It is noteworthy that the mechanisms of action for approximately 18% of approved therapeutic drugs at present, including CLZ, remain unknown [11,12,13]. CLZ is considered the last pharmacological option to treat refractory psychosis, thus knowledge of its mechanisms of action will help to improve patient treatment and drug repositioning [14,15]. Among the strategies for pharmacological repositioning, the omics approach of biological data has provided integrative data through computational and statistical methods [14,16,17,18].

The plasma concentrations (lower range of utility = 250–400 ng/mL) [7] and metabolic ratios of CLZ are broadly related to the prescribed dose, exhibiting a great variability between individuals. The metabolic ratio is calculated as the ratio of unmetabolized drug to its main metabolite, *N*-desmethylclozapine or norclozapine (NCLZ), in plasma samples [19] and is optimally defined as approximately two [20]. Other CLZ-associated phenotypes of interest that should be evaluated during its prescription are dosage and response. CLZ dosage is controversial in terms of clinical response, effectiveness, and the presence of side effects, and although several exploratory studies have been carried out in this regard the relationship still remains unclear. Despite the wide variation in CLZ dosage in clinical practice, there is a consensus that doses below 100 mg may be insufficient for patients to respond to, thus the standard dose is usually between 300 and 600 mg [21,22,23]. In this context, an integrative omics data analysis of patients with refractory psychosis would be of aid in identifying markers to improve or predict some of the CLZ-associated phenotypes (i.e., metabolic ratio, dosage, and response). 

The high interindividual variability of CLZ-associated phenotypes is due to interactions between nongenetic, genetic, and epigenetic factors [8,24]. Genome-wide studies of psychosis have explored polygenic risk scores (PRS), showing that most disorders associated with psychosis share a genetic basis [25]. Moreover, when comparing individuals with a high PRS vs. individuals with a low PRS, a positive correlation between PRS and DNA methylation changes has been observed (the higher the PRS, the greater the methylation changes) [26]. 

Herein, we present an integration of clinical, genomic, and epigenomic data from CLZ-treated patients with refractory psychosis in order to identify genes related to the potential mechanisms of action of CLZ and its possible pharmacogenomics applications.

## 2. Results

### 2.1. Clinical and Demographic Characteristics of Patients

Table 1 shows the clinical and demographic characteristics of CLZ-treated patients. A total of 75% of our patients were taking concomitant medications.

### 2.2. Association Between Genetic Risk Scores and Clozapine-Associated Phenotypes 

**Figure 1:**
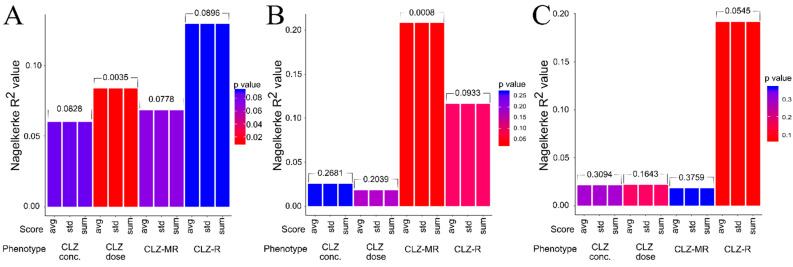
Results of polygenic risk score (PRS) analysis for major depressive disorder (**A**), bipolar disorder (**B**), and schizophrenia (**C**) and their associations with clozapine-associated phenotypes. The *X*-axis contains the clozapine-associated phenotypes and the *Y*-axis shows the proportion of variance explained by the PRS that was calculated by Nagelkerke’s pseudo-R^2^ value. The colors of the bars are in accordance with the associated *p*-values (e.g., the redder the bar, the more significant the *p*-value). Abbreviations: CLZ, clozapine; conc, concentration; MR, metabolic ratio; R, response; avg, average; std, standard.

After the samples were genotyped using the Illumina Infinium PsychArray v1.2 BeadChip, we calculated the PRSs for schizophrenia (SZ-PRS), bipolar disorder (BD-PRS), and major depressive disorder (MDD-PRS). Two nominal associations were observed between PRS and CLZ-associated phenotypes—namely, MDD-PRS with the CLZ dose (pseudo-R^2^ = 0.386, *p*-value = 0.0035) and SZ-PRS with the response to CLZ (pseudo-R^2^ = 0.191, *p*-value = 0.0545); however, they did not remain significant after adjustment for multiple comparisons (adjusted *p*-values = 0.0759 and 0.2278, respectively) (Figure 1). The only PRS that showed a significant association with any CLZ-related phenotype was the BD-PRS. The BD-PRS was associated with the CLZ metabolic ratio (pseudo-R^2^ = 0.2080, *p*-value = 0.0008, adjusted *p*-value = 0.0189).

### 2.3. Functional Prediction of the SNPs Included in the BD-PRS Associated with CLZ Metabolic Ratio

The BD-PRS associated with the CLZ metabolic ratio was constituted by 2112 single-nucleotide polymorphisms (SNPs), of which 1288 were located in intronic regions, 223 in exonic regions, and 562 in intergenic regions. The SNPs that made up the BD-PRS were found in 1370 genes. These genes were the top enriched in four pathways: circadian rhythms (*ADCY2, CACNA1C, CACNA1D, MAPK1*), insulin secretion (*ABCC8, ADCY9, ATP1B2, KCNMA1*), GABAergic synapse *(CACNA1B, GABRA1, KCNJ6, SLC12A5)*, and the thyroid hormone signaling pathway *(AKT3, ATP1B3, RXRA, TP53)* (Table 2). We found a total of 17 SNPs that could have a high impact on the protein structure in genes such as *LRP8* and *ADCY2*, among others (Supplementary Tables S1–S3).

### 2.4. Differentially Methylated Sites Between Patients Grouped by BD-PRS and CLZ Metabolic Ratios

**Figure 2:**
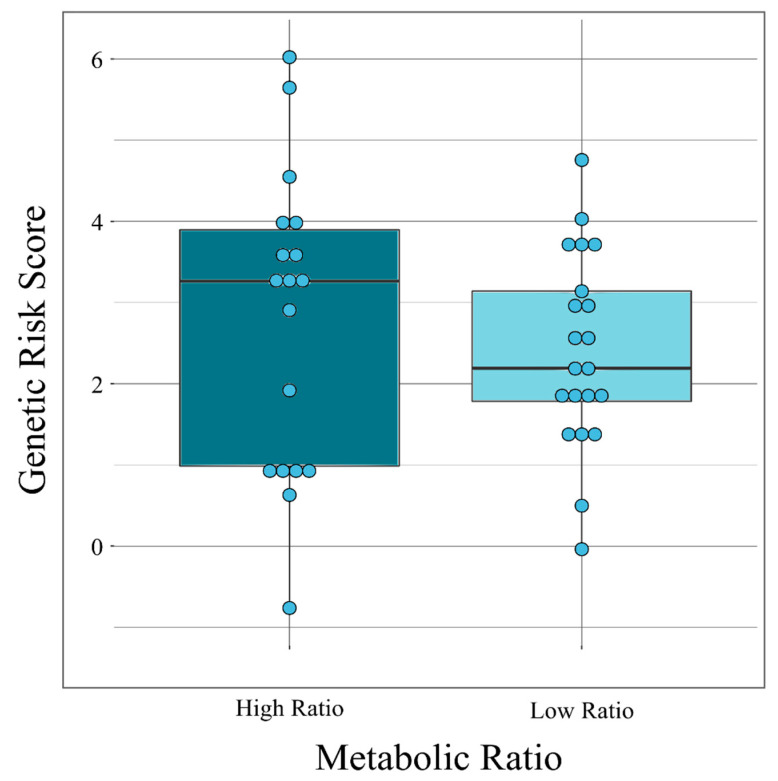
Subgroups of clozapine-treated patients according to their metabolic ratios and bipolar disorder genetic risk score. Box plot showing the distribution of values and the cut-off points for the metabolic ratios and genetic risk scores.

In order to explore whether BD-PRSs associated with the CLZ metabolic ratio could alter DNA methylation patterns, we evaluated the differential methylation using the Infinium MethylationEPIC array in subgroups of CLZ-treated patients according to their metabolic ratios (CLZ/NCLZ) and BD-PRS values. The cut-off point for the metabolic ratio was defined as 2.0 according to published recommendations [7], and the medians were 3.2639 and 2.1922 for the high and medium BD-PRS cut-off points, respectively. Thus, samples with a metabolic ratio < 2.0 or ≥ 2.0 were assigned a low or high metabolic ratio, respectively. Accordingly, the following three groups were obtained for the BD-PRSs (Figure 2): samples with a high metabolic ratio and a high BD-PRS (HH ≥ 3.2639), a medium BD-PRS (M) for values <3.2639 but > 2.1922, and a low BD-PRS (L) of ≤2.1922.

In the comparison between these subgroups (HH vs. M, HH vs. L, M vs. L) regarding the differential methylation analysis, the associations were not statistically significant at the genome-wide level (*p*-value < 5.0 × 10^−8^). We observed nominal associations after comparing the HH vs. M (in three CpG sites), and M vs. L groups (in three different CpG sites) (Table 3 and Table S2). No significance was found between the HH and L groups.

CpG sites with a nominal association (*p*-value < 5.0 × 10^−5^) between the H and M groups were located on the *TESPA1* and *APOB* genes. The CpG site for *TESPA1* (cg23612423) was hypomethylated in the H group, whereas the CpG site for *APOB* (cg16723488) was hypermethylated in the M group. CpG sites with a nominal association between the M and L groups were located on the *APOB* (cg05337441) and *STAG1* (cg16760310) genes. Both genes were hypermethylated in the M group. In contrast, the CpG site at *FUOM* (cg05456948) was hypomethylated in the same group (Table 3). 

### 2.5. Protein–Protein Interactions Between Gene Products with High Impact Variants in the Top Enriched Pathways and Differentially Methylated Sites

**Figure 3:**
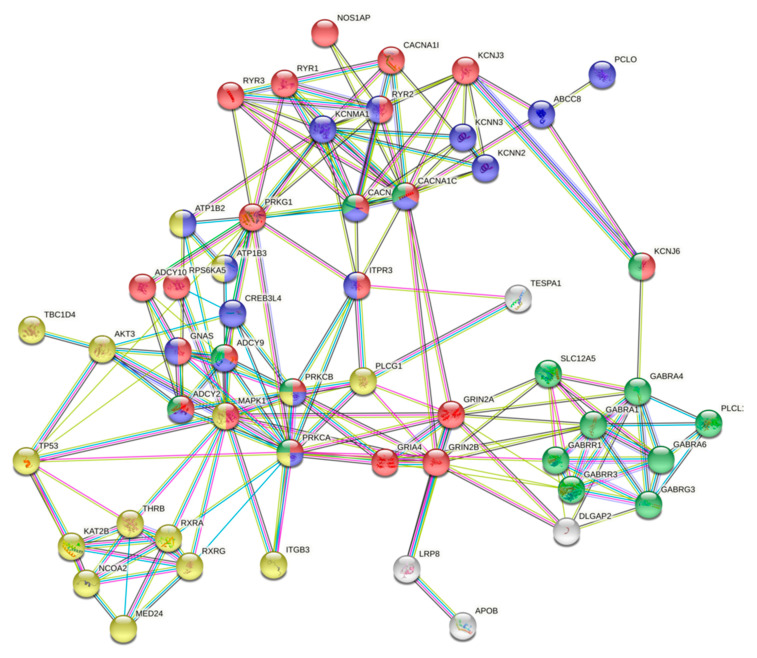
Protein–protein interaction network between gene products with high-impact variants contained in the top enriched pathways and those presenting differentially methylated sites in CLZ-treated patients. Proteins are grouped by colors according to the pathway they are involved in (e.g., proteins in red: circadian entrainment; blue: insulin secretion; yellow: thyroid hormone signaling pathway; green: GABAergic synapse). Clusters are based on STRING analysis. The center of the figure highlights the close interactions between nodes.

A second pathway enrichment analysis was carried out, but this time the protein–protein interactions included genes products of: (i) BD-PRSs showing variants with a high functional impact, (ii) previous enriched pathways, and (iii) differentially methylated genes between the three BD-PRS groups (Figure 3). 

This analysis revealed multiple interactions. For instance, *APOB* (a gene with differentially methylated sites) interacts strongly with *LRP8* (a gene that contains the missense variant p.Arg952Gln), which, in turn, interacts with genes enriched in the circadian rhythm pathway (e.g., *GRIN2B, GRIN2A*, and *GRIA4*). Two of the aforementioned genes (*GRIN2B* and *GRIN2A*) also interact with genes involved in the GABAergic synapse (i.e., *GABRR1, GABRR3*, and *GABRA1*) and with *DLGAP2* (a gene that shows the missense variant p.Pro464Gln). Interestingly, *GRIA4* interacts with *PRKCB* and *PRKCA*, and both genes are included in the BD-PRS and are enriched in the top four observed canonical pathways—namely, circadian entrainment, insulin secretion, GABAergic synapse, and the thyroid hormone signaling pathway. Moreover, the *PRKCA* and *PRKCB* genes interrelate with *ADCY2* (a gene that contains the missense variant p.Val147Met), which in turn interconnects with *PLCG1* and links with *TESPA1* (this gene shows differentially methylated sites) (Table 3 and Table S1).

## 3. Discussion

Overall, CLZ has been utilized as an antipsychotic drug due to its simultaneous affinity for both dopamine and serotonin receptors [28]. Nonetheless, its complex mechanisms of action are not yet fully known, involving the modulation of norepinephrine, the regulation of the endocrine system (including pregnenolone and cortisol), the intracellular system-dependent modulation of *N*-methyl-d-aspartate (NMDA) receptor expression, brain-derived neurotrophic factor up-regulation, and the regulation of the arachidonic acid cascade [29,30,31,32]. Herein, we performed an integration of the genomic and epigenomic data of CLZ-associated phenotypes to identify genes related to the potential mechanisms of action of CLZ and possible pharmacogenomic applications. First, we identified that the BD-PRSs were associated with the CLZ metabolic ratios. The CLZ/NCLZ ratio may be interpreted as the rate of hepatic metabolism of the antipsychotic administered orally (as is the case). Consequently, the higher the ratio the lower the metabolism in the liver [7]. This result might be related to the SNPs contained in the BD-PRS (Table 2), which were enriched in the insulin secretion pathway and the thyroid hormone signaling pathway (Table S1).

The thyroid hormone signaling pathway is activated by the consumption of glucose-rich foods [33], mainly through Ca^2+^ currents that are modulated by channels such as CACNA1C or CACNA1D (genes found in the BD-PRS) [34,35,36]. It is known that individuals with BD and psychosis have an increased risk of diabetes mellitus (i.e., high blood glucose levels) [37,38,39]. In fact, it has been reported that CLZ response and diabetes mellitus share genetic mechanisms [40,41,42], including recurrent genes such as *CACNA1C* in common pathways (e.g., insulin secretion). Additionally, it has been documented that hyperglycemia may reduce the response to the mood stabilizer treatments [43]. This reduction may be due to a long-term consequence of hyperglycemia disrupting the hepatic expression of genes involved in pharmacological metabolization [44,45,46]. Besides the effects that hyperglycemia could have in CLZ-treated patients with refractory psychosis, we also identified relevant gene enrichment in the thyroid hormone signaling pathway, including the *RXRA/RXRG* genes. The retinoid X receptors (RXRA and RXRG) are considered xenobiotic sensors that may induce the expression of the cytochrome P450 system [47,48,49]. In this sense, the induction of cytochrome P450 enzymes would promote an increase in the CLZ metabolism; however, if an increase in deleterious genetic variants affecting that pathway exists (as shown in this study), then it will diminish the induction of CLZ-DMEs, and its metabolic ratio will increase. An interesting finding that could be related to this effect of an increase in risk variants in *RXRA/RXRG* is the observed hypomethylation in *APOB* in patients of the HH group. PPARs, together with retinoid X receptors (RXRs), regulate the transcription of *APOB* and *APOE*, among others [50,51,52]. In this context, the hypomethylation of *APOB* could increase the gene expression in group HH patients as a mechanism of compensation for the pathway dysfunction due to the increase in risk variants in genes from the retinoic acid pathway [53,54,55]. 

We also found that the *LRP8* variant p.Arg952Gln (rs5174), which was included in the BD-PRS, shows a high functional impact, and it has previously been associated with psychosis [56]. The encoded protein, LRP8, is a receptor of RELN (whose abnormal expression is associated with major neuropsychiatric disorders), but also functions as a receptor for the cholesterol transport protein APOE [57]. It is known that the hepatic APOE levels increase during CLZ treatment, as well as other genes participating in the transport of cholesterol; however, if this *LRP8* variant promotes a decrease in the receptor function [58,59], one can hypothesize that the hypomethylation of *APOB* could also be a compensatory mechanism for this decrease [60,61]. 

The identified relationship between *APOB* and *LRP8* points towards an association of CLZ with glutamatergic regulation. The receptor LRP8 interacts with NMDA receptor subtypes 2B and 2A (GRIN2B and GRIN2A), thereby mediating reelin signaling [61,62]. NMDA receptors are generally located next to glutamatergic and GABAergic vesicles [63]. Interestingly, the GABAergic synapse pathway was found to be enriched in BD-PRS and was associated with the CLZ/NCLZ ratio. This complex association of the GABAergic synapse with BD-PRS and the metabolic ratio poses the question of whether CLZ could be used as a mood stabilizer and not just as an antipsychotic. Indeed, GABAergic dysfunction has been considered as a hypothesis of mood disorders [64]. This hypothesis was proposed after treatment with valproate showed efficacy in BD patients, thus becoming the most widely used mood stabilizer. CLZ, although not an approved drug for the treatment of BD, has been used with some improvement in individuals with resistance to treatment or in severe cases of mania [65,66]. Considering that CLZ might have some effect in stabilizing mood, it should bind to GABAergic receptors. CLZ generally binds to dopamine and serotonergic receptors; however, its binding to GABAergic receptors is still being explored. Two studies in animal models have demonstrated that acute CLZ treatment induces epigenetic changes in the GABAergic gene promoters [57,67,68]. In a molecular docking study, the authors found that CLZ could bind to the receptor GABABR in the same manner as baclofen does (an agonist of GABABR) [69]. Herein, the GABAergic synapse was found to be enriched in BD-PRS and associated with the metabolic ratio, supporting the potential effect of CLZ in the GABAergic synapse.

Another point that may support the use of CLZ as a mood stabilizer is the fact that many BD-PRS variants are found in calcium-dependent genes (*CACNA1C* and *CACNA1D*). *CACNA1C* is one of the genes that has been associated with BD in both genome- and epigenome-wide association studies [70,71], modulating the cerebral cortex and hippocampus function [72,73]. *CACNA1C* is generally hypermethylated in BD patients [71], and these DNA methylation changes may depend on genetic variants close to the gene locus. 

Finally, in the protein–protein interaction analysis, we identified that CACNA1C interacts with ITPR3, which, in turn, interacts with TESPA1. Their corresponding genes were included in BD-PRS and were found to be differentially methylated, respectively. ITPR3 is the inositol 1,4,5-trisphosphate receptor type 3, a second messenger that mediates the release of intracellular calcium with ubiquitous expression [74,75,76]. ITPR3 and TESPA1 interaction regulates calcium flux and modulates different immune system functions [77,78,79]. In this sense, we found that individuals with a high metabolic ratio and high BD-PRS presented hypermethylation in *TESPA1*, which may be promoted by an increase in Ca^2+^ signaling due to the accumulation of deleterious variants in the calcium pathway (such as those present in *CACNA1C* and *ITPR3*).

We included patients with psychosis that met the clinical criteria of SZ, BD, or SD, but we should consider that these disorders constitute a well-recognized clinical spectrum. In relation to this, there are clinical and epidemiological studies considering SZ and BD as a single major psychosis phenotype, demonstrating the shared genetic liability and overlapping polygenic component of the two illnesses [80,81]. SD has been less investigated but shows substantial familial overlap with both SZ and BD [82]. The pharmacogenomics of the antipsychotics response in this clinical spectrum has mainly focused on the study of genetic variants associated with pharmacokinetics, whereas pharmacodynamics has not been explored in detail. In this regard, the obligated phenotypes for evaluation are SZ-associated genes [83,84]. In the present study, we analyzed BD-PRS, SZ-PRS, and MDD-PRS, and no association with CLZ response was found; however, evaluating other associated genes, even when they are not within the strict diagnostic criteria, becomes of some importance.

The findings of this study feature some limitations. First, the small sample size and lack of patient biochemical data (e.g., lipid profiles) prevented us from further exploring our results regarding other clinical variables. Second, because we used peripheral tissue, some of our results might not be the same as those observed at the brain level (e.g., *TESPA1*, a gene with differentially methylated sites, is highly expressed in leukocytes but not in the brain). Third, we cannot rule out the possibility of other unidentified associations in these samples, since we only analyzed the genes included in the microarrays we used. Fourth, all the CLZ-treated patients included here had refractory psychosis, even though their clinical diagnoses were different (SZ, SD, or BD), which might have affected the estimation ability of our study (statistical power = 70%, calculated with the Graw algorithm for the relationship between DNA methylation and CLZ-associated phenotypes) [85]. Thus, future studies with larger sample sizes should consider the inclusion of these missing elements.

This study pioneers the exploration of genomics and methylomics simultaneously in Mexican patients with psychosis in the context of CLZ treatment. Our results suggest the use of CLZ as a mood stabilizer, primarily in the treatment of psychosis. Furthermore, we present methods integrating both omic technologies to better characterize the pharmacogenomics of clozapine.

## 4. Materials and Methods 

### 4.1. Patients

Forty-four unrelated patients with refractory psychosis (unresponsive to at least two previous antipsychotic treatments) were consecutively recruited from the outpatient service at the National Institute of Neurology and Neurosurgery “Manuel Velasco Suárez” (NINN) in Mexico City. The inclusion criteria were patients with at least the two previous generations having been born and brought up in Mexico and those with a Spanish surname. The clinical diagnosis of SZ, SD, or BD was carried out based on the DSM-5 criteria [86] and was performed by at least one psychiatrist specialized in psychotic disorders. All the patients experienced CLZ monotherapy as an antipsychotic treatment for more than 18 weeks. The exclusion criteria were neurologic disease, heavy drinkers and/or heavy smokers, substance abuse within the past 6 months, history of a head injury with a loss of consciousness greater than 5 min or with documented neurocognitive sequelae, intellectual disability, trauma in general, and medical illnesses that may be associated with significant neurocognitive impairment.

This study was carried out in accordance with the latest version of the Declaration of Helsinki and was approved by the local research and ethical committees (protocol NINN_104/17, amended in 2018). Written informed consent was obtained from all participants after the nature of the procedures had been fully explained. 

### 4.2. Clozapine and Norclozapine Plasma Concentrations.

Blood samples were taken at steady state (i.e., at week 18 of treatment). The preparation of plasma samples was carried out as previously reported [27], and plasma concentrations of CLZ and its main metabolite (ng/mL), *N*-desmethylclozapine or nor-clozapine (NCLZ), were determined by HPLC, and the metabolic ratios of CLZ/NCLZ were also calculated. 

### 4.3. Analysis and Quality Control of Microarrays

Blood DNA was isolated by standard procedures after 18 weeks of treatment under CLZ. The samples were genotyped using the Infinium PsychArray v1.2 BeadChip (Illumina, San Diego, CA, USA) and then imputed. The genome-wide DNA methylation levels were measured using the Infinium MethylationEPIC BeadChip (Illumina, San Diego, CA, USA). The Genome Reference Consortium Human Build 37 (GRCh37/hg19) was used for all the analyses.

DNA samples were hybridized with PsychArray according to the manufacturer’s instructions and scanned on an iScan Microarray Scanner (Illumina). The genotypes obtained with GenomeStudio (Illumina) were filtered for quality control following the PLINK v.6.21 program criteria [87]. Thus, we discarded genetic variants and samples with either a variant calling <95%, a minor allele frequency (MAF) < 0.05 (as reported in the 1000 Genomes Project), and variants that were not in Hardy–Weinberg equilibrium using a chi-square method with a value of *p* < 1 × 10^−6^. For the epigenomic analysis, DNA was bisulfite-converted (Zymo, Irvine, CA, USA) and hybridized to EPIC while following the manufacturer’s protocol. The fluorescence intensities were measured with the iScan instrument and transformed into idat files with the algorithm implemented in the GenomeStudio. Raw methylation data were filtered out using the following criteria in the ChAMP package [88]: detection of *p*-value > 0.01, probes with less than 3 beads in <5% of the samples, probes located on sites not-CpGs or associated with SNPs, sex chromosome probes, multihit probes, and probes with rates greater than 0.1 were removed. After performing the quality control, 741,030 probes remained, and a matrix of beta values was built including the 44 patients. The matrix was adjusted for the differences in cell proportions by a deconvolution method in the ChAMP package. Genotyping and microarray analyses were carried out by specialized staff in the Microarray Unit of the National Institute of Genomic Medicine Mexico City, Mexico (INMEGEN).

### 4.4. Analysis of Polygenic Risk Score

To calculate the polygenic risk score (PRS) for SZ, BD, and major depressive disorder (MDD), we used the latest available GWAS summary statistics from the Psychiatric Genomics Consortium (i.e., SZ-PRS was derived from PGC wave-2 group, BD-PRS was calculated using BIP2018 dataset, and MDD-PRS was generated from results of the PGC GWAS and 23 and Me) as a training set [89,90,91] and our genotyped sample as the target. Poisson correlations were used to test the associations between PRS and CLZ-associated phenotypes—namely, disease improvement (CLZ response and non-response), the dose of CLZ, the CLZ plasma concentrations, and the metabolic ratios (CLZ/NCLZ). Depending on the studied phenotype, logistic or linear regressions were performed with PRSice v.2.3.3 [92]. PRSice uses two steps to construct the PRS. First, there is the clumping process, where SNPs in linkage disequilibrium (LD) between the associated loci in the target sample and the discovery sample are unified. Second, PRSice calculates the individual PRS using different *p*-value thresholds for the associated variants in the discovery sample, thereby calculating the best-fit PRS for the target sample (starting with a *p*-value threshold of 0.5 from the GWAS with increments of 0.00005). The best-fit model for the PRS explains the greatest amount of variance in the phenotype by estimating Nagelkerke’s pseudo-R^2^ value. We considered an association between PRS and CLZ phenotypes after 1000 permutations to correct for multiple tests when the *p*-value was less than 0.05. Additionally, all the regressions were adjusted for age, gender, and the 10 main components of global ancestry. Global ancestry estimation was performed with the PC-AiR package [93] using the reference panel of the Human Genome Diversity Project.

### 4.5. Analysis of Differentially Methylated Regions (DMRs)

The methylation patterns among groups were evaluated by linear models implemented in the limma package [94], and the statistically significant *p*-value was <1 × 10^−8^. 

### 4.6. Functional Annotation and Pathway Enrichment Analysis

The SNPs integrating the PRS were annotated using the Variant Effect Predictor (VEP, web version) using the human genome reference assembly hg19. The VEP is a toolset for the analysis, annotation, and prioritization of genomic variants that predicts the functional effect of variants in silico using different databases and prediction algorithms (CADD, SIFT, PolyPhen2, and LoF) [95]. We classified SNPs based on their coding positions in exonic and non-exonic variants. Exonic variants were filtered out if they were predicted as deleterious in SIFT, and possibly or probably damaging in Polyphen. A CADD value higher than 25 was used for non-exonic variants. The functional enrichment analysis of the PRS genes and differentially methylated genes was carried out by WebGestalt [96]. In addition, the protein interaction analysis was carried out using STRING [97].

## 5. Conclusions

Our study is the first to show simultaneous genomic–epigenomic signatures in samples from patients with refractory psychosis and those under CLZ treatment, which raises several questions regarding the genetic/epigenetic determinants of BD-PRS for CLZ-associated phenotypes and opens up many avenues for future studies. We strongly believe that these are important results for this field.

## Figures and Tables

**Table 1:** Clinical and demographic characteristics of clozapine-treated patients (*n* = 44).

Characteristic	Number (%) or Mean ± Standard Deviation
Clinical diagnosis	
Schizophrenia	31 (70.45%)
Schizoaffective disorder	9 (20.45%)
Bipolar disorder	4 (9.09%)
Number of Male Patients (%)	28 (63.60%)
Age (years)	37.40 ± 11.30
Age at onset	18.50 ± 9.80
School (Years)	13.30 ± 2.90
Number of patients who are smokers (%)	22 (50.00%)
Number of patients who are drinkers (%)	13 (29.50%)
CLZ Dose (mg/day)	202.60 ± 138.02
CLZ responders	36 (81.80%)
CLZ and its metabolite determinations	
* Plasma concentrations of CLZ (ng/mL)	154.03 ± 191.97

CLZ: clozapine; NCLZ: norclozapine. * Determined by HPLC [27].

**Table 2:** Functional single-nucleotide polymorphisms (SNPs) with a possible high impact on the polygenic risk score for bipolar disorder and clozapine metabolic ratios.

Location ^†^	Gene Symbol	Gene Name	Genetic Variant ID	Minor Allele Frequency	Protein ID	Variant Location in Coding Region
chr1:53712727	*LRP8*	LDL receptor-related protein 8	rs5174	T = 0.204	NP_004622.2:p.Arg952Gln	Missense variant
chr1:151374025	*PSMB4*	Proteasome 20S subunit beta 4	rs4603	C = 0.273	NP_002787.2:p.Ile234Asn	Missense variant
chr1:151733335	*MRPL9*	Mitochondrial ribosomal protein L9	rs8480	G = 0.443	NP_113608.1:p.Glu210Val	Missense variant
chr4:162307312	*FSTL5*	Follistatin-like 5	rs3749598	A = 0.216	NP_064501.2:p.Asp711Tyr	Missense variant
chr5:7520768	*ADCY2*	Adenylate cyclase 2	rs13166360	T = 0.057	NP_065433.2:p.Val147Met	Missense variant
chr5:898209847	*LYSMD3*	LysM domain containing 3	rs10069050	C = 0.375	NP_938014.1:p.Glu41Asp	Missense variant
chr6:142396790	*NMBR*	Neuromedin B receptor	rs7453944	T = 0.307	NP_002502.2:p.Leu390Met	Missense variant
chr7:64439701	*ZNF117*	Zinc finger protein 117	rs3807069	T = 0.307	NP_056936.2:p.Cys83Tyr	Missense variant
chr7: 92733766	*SAMD9*	Sterile alpha motif domain-containing 9	rs10279499	A = 0.091	NP_001180236.1:p.Val549Leu	Missense variant
chr7:104717517	*KMT2E*	Lysine methyltransferase 2E (inactive)	rs2240455	T = 0.216	NP_061152.3:p.Tyr292Ter	* Stop_gained
chr7:129663496	*ZC3HC1*	Zinc finger C3HC-type containing 1	rs11556924	T = 0.148	NP_057562.3:p.Arg363His	Missense variant
chr8:1514009	*DLGAP2*	DLG associated protein 2	rs2301963	C = 0.284	NP_001333739.1:p.Pro464Gln	Missense variant
chr12:108618630	*WSCD2*	WSC domain containing 2	rs3764002	T = 0.125	NP_055468.2:p.Thr266Ile	Missense variant
chr15:84639350	*ADAMTSL3*	ADAMTS-like 3	rs2277849	T = 0.189	NP_997400.2:p.Leu869Phe	Missense variant
chr16:3639827	*SLX4*	SLX4 structure-specific endonuclease subunit	rs3810813	A = 0.079	NP_115820.2:p.Ser1271Phe	Missense variant
chr17:35988672	*DDX52*	DExD-box helicase 52	rs7224513	C = 0.239	NP_008941.3:p.Arg264Ser	Missense variant
chr17:73513677	*TSEN54*	tRNA splicing endonuclease subunit 54	rs11559205	C = 0.091	NP_997229.2:p.Ile137Leu	Missense variant

^†^ Physical location of the gene (hg19). Genetic variant and protein identifiers (ID) according to the Single Nucleotide Polymorphism Database (dbSNP) and the protein database at the National Center for Biotechnology Information (NCBI). * Combined Annotation Dependent Depletion (CADD) prediction score = 35.

**Table 3:** Differentially methylated regions in DNA samples according to their bipolar disorder-PRS and clozapine metabolic ratios.

Location ^†^	Gene Symbol	CpG Site	Feature	Location Relative to cgi	LogFC	Avg Methylation			*p*-Value	
						High PRS	Medium PRS	Low PRS	High-Medium PRS	Medium-Low PRS
	*TESPA1*	cg23612423	3’UTR	Open sea	−0.14346761	0.5651155	0.42164789	0.52374574	9.06 × 10^−7^	4.01 × 10^−2^
chr2:21266669-21266961	*APOB*	cg16723488	TSS200	Island	0.09815776	0.37368773	0.4718455	0.39797591	8.38 × 10^−6^	2.42 × 10^−5^
chr2:21266669-21266961	*APOB*	cg05337441	Body	Shore	0.08863618	0.15337978	0.2555618	0.16692562	2.46 × 10^−5^	3.02 × 10^−6^
chr8:58055960-58056244	*-*	cg11062466	IGR	Shore	0.27464151	0.30018264	0.57482415	0.36696224	8.92 × 10^−6^	6.11 × 10^−3^
chr10:135170645-135171954	*C10orf125*	cg05456948	TSS200	Island	−0.04000716	0.19107189	0.15466524	0.1946724	3.04 × 10^−04^	1.54 × 10^−06^
	*STAG1*	cg16760310	Body	Open sea	0.02946489	0.932180391	0.96574714	0.93628225	1.09 × 10^−3^	7.21 × 10^−6^

^†^ Physical location of the gene (hg19). CGI, CpG island. FC, fold-change. Avg, average. PRS, bipolar disorder-polygenic risk score. Chr, chromosome. UTR, untranslated region. TSS, transcription start site. IGR, intergenic region.

## Data Availability

The data presented in this study are available in Supplementary material. Additional data are available on request from the corresponding author due to privacy and ethical issues.

## Supplementary Materials

The following files are available online https://www.mdpi.com/1424-8247/14/2/118/s1: Table S1: Table summarizing the top enriched molecular pathways of the genes included in the bipolar disorder polygenic risk score and associated to clozapine metabolic ratios; Table S2: The contrast matrix; Table S3: The complete lists of SNPs for each CLZ-associated phenotype.
