# Supplementary material for: Integrative Genomic–Epigenomic Analysis of Clozapine-Treated Patients with Refractory Psychosis
Source: Pharmaceuticals (Basel). 2021 Feb 4;14(2):118. doi: 10.3390/ph14020118 (PMC7913835; doi:10.3390/ph14020118)
Supplement: Supplementary file 1 [file pharmaceuticals-14-00118-s001.zip › PHarmaceuricals 1050486 Supplementary/Supplementary Table S1_Mayen-Lobo YG et al..docx]

SUPPLEMENTARY MATERIALS

Integrative genomic-epigenomic analysis of clozapine-treated patients with refractory psychosis

Yerye Gibrán Mayén-Lobo^1,2^, José Jaime Martínez-Magaña^3^, Blanca Estela Pérez-Aldana^1^, Alberto Ortega-Vázquez^1^, Alma Delia Genis-Mendoza^3^, David José Dávila-Ortiz de Montellano^2^, Ernesto Soto-Reyes^4^, Humberto Nicolini^3,5^, Marisol López-López^1^, and Nancy Monroy-Jaramillo^2,^*

^1^ Department of Biological Systems, Metropolitan Autonomous University-Xochimilco. Mexico City, Mexico; [yeryegibran@gmail.com](mailto:yeryegibran@gmail.com) (Y.G.M.L.); [blankita0807@gmail.com](mailto:blankita0807@gmail.com) (B.E.P.A.); [betoov@yahoo.com.mx](mailto:betoov@yahoo.com.mx) (A.O.V.); [mlopez@correo.xoc.uam.mx](mailto:mlopez@correo.xoc.uam.mx) (M.L.L.)

^2^ Department of Genetics. National Institute of Neurology and Neurosurgery, “Manuel Velasco Suárez”. Mexico City, Mexico; [djdodem@gmail.com](mailto:djdodem@gmail.com) (D.J.O.M.)

^3^ Genomics of Psychiatric and Neurodegenerative Diseases Laboratory, Instituto Nacional de Medicina Genómica, SSA, Mexico City, Mexico; [jimy.10.06@gmail.com](mailto:jimy.10.06@gmail.com) (J.J.M.M.); [adgenis@inmegen.gob.mx](mailto:adgenis@inmegen.gob.mx) (A.D.G.M.)

^4^ Natural Sciences Department, Universidad Autónoma Metropolitana-Cuajimalpa, Mexico City, Mexico; [esotoreyes@cua.uam.mx](mailto:esotoreyes@cua.uam.mx) (E.S.R.)

^5^ Grupo de Estudios Médicos y Familiares Carracci, Mexico City, Mexico; [hnicolini@inmegen.gob.mx](mailto:hnicolini@inmegen.gob.mx) (H.N.)

***** Correspondence: [nancy97@unam.mx](mailto:nancy97@unam.mx); Tel.: +52-55-5606-3822.

Index.

Supplementary Materials Table S1. Summary of the top enriched molecular pathways of the genes included in the bipolar disorder polygenic risk score and associated to clozapine metabolic ratios

**Supplementary Table 1**. Top enriched molecular pathways of the genes included in the bipolar disorder polygenic risk score and associated to clozapine metabolic ratios

| **Pathway** | **Index** | **p-value** | **FDR** | **Gene symbol** | **Gene name** |
| --- | --- | --- | --- | --- | --- |
| Circadian entrainment | hsa04713 | 1.9395e-8 | 5.8631e-6 | *ADCY10* | Adenylate cyclase 10 |
|  |  |  |  | ***ADCY2*** | Adenylate cyclase 2 |
|  |  |  |  | ***ADCY9*** | Adenylate cyclase 9 |
|  |  |  |  | ***CACNA1C*** | Calcium voltage-gated channel subunit alpha1 C |
|  |  |  |  | ***CACNA1D*** | Calcium voltage-gated channel subunit alpha1 D |
|  |  |  |  | *CACNA1I* | Calcium voltage-gated channel subunit alpha1 I |
|  |  |  |  | ***GNAS*** | GNAS complex locus |
|  |  |  |  | *GRIA4* | Glutamate ionotropic receptor AMPA type subunit 4 |
|  |  |  |  | *GRIN2A* | Glutamate ionotropic receptor NMDA type subunit 2A |
|  |  |  |  | *GRIN2B* | Glutamate ionotropic receptor NMDA type subunit 2B |
|  |  |  |  | ***ITPR3*** | Inositol 1,4,5-trisphosphate receptor type 3 |
|  |  |  |  | *KCNJ3* | Potassium voltage-gated channel subfamily J member 3 |
|  |  |  |  | ***KCNJ6*** | Potassium voltage-gated channel subfamily J member 6 |
|  |  |  |  | ***MAPK1*** | Mitogen-activated protein kinase 1 |
|  |  |  |  | *NOS1AP* | Nitric oxide synthase 1 adaptor protein |
|  |  |  |  | ***PRKCA*** | Protein kinase C alpha |
|  |  |  |  | ***PRKCB*** | Protein kinase C beta |
|  |  |  |  | *PRKG1* | Protein kinase cGMP-dependent 1 |
|  |  |  |  | *RPS6KA5* | Ribosomal protein S6 kinase A5 |
|  |  |  |  | *RYR1* | Ryanodine receptor 1 |
|  |  |  |  | *RYR2* | Ryanodine receptor 2 |
|  |  |  |  | *RYR3* | Ryanodine receptor 3 |
| Insulin secretion | hsa04911 | 6.0260e-6 | 3.9289e-4 | *ABCC8* | ATP binding cassette subfamily C member 8 |
|  |  |  |  | ***ADCY2*** | Adenylate cyclase 2 |
|  |  |  |  | ***ADCY9*** | Adenylate cyclase 9 |
|  |  |  |  | ***ATP1B2*** | Atpase Na+/K+ transporting subunit beta 2 |
|  |  |  |  | ***ATP1B3*** | Atpase Na+/K+ transporting subunit beta 3 |
|  |  |  |  | ***CACNA1C*** | Calcium voltage-gated channel subunit alpha1 C |
|  |  |  |  | ***CACNA1D*** | Calcium voltage-gated channel subunit alpha1 D |
|  |  |  |  | *CREB3L4* | Camp responsive element binding protein 3 like 4 |
|  |  |  |  | ***GNAS*** | GNAS complex locus |
|  |  |  |  | ***ITPR3*** | Inositol 1,4,5-trisphosphate receptor type 3 |
|  |  |  |  | *KCNMA1* | Potassium calcium-activated channel subfamily M alpha 1 |
|  |  |  |  | *KCNN2* | Potassium calcium-activated channel subfamily N member 2 |
|  |  |  |  | *KCNN3* | Potassium calcium-activated channel subfamily N member 3 |
|  |  |  |  | *PCLO* | Piccolo presynaptic cytomatrix protein |
|  |  |  |  | ***PRKCA*** | Protein kinase C alpha |
|  |  |  |  | ***PRKCB*** | Protein kinase C beta |
|  |  |  |  | *RYR2* | Ryanodine receptor 2 |
| GABAergic synapse | hsa04727 | 3.9474e-5 | 1.6086e-3 | ***ADCY2*** | Adenylate cyclase 2 |
|  |  |  |  | ***ADCY9*** | Adenylate cyclase 9 |
|  |  |  |  | *CACNA1B* | Calcium voltage-gated channel subunit alpha1 B |
|  |  |  |  | ***CACNA1C*** | Calcium voltage-gated channel subunit alpha1 C |
|  |  |  |  | ***CACNA1D*** | Calcium voltage-gated channel subunit alpha1 D |
|  |  |  |  | *GABRA1* | Gamma-aminobutyric acid type A receptor alpha1 subunit |
|  |  |  |  | *GABRA4* | Gamma-aminobutyric acid type A receptor alpha4 subunit |
|  |  |  |  | *GABRA6* | Gamma-aminobutyric acid type A receptor alpha6 subunit |
|  |  |  |  | *GABRG3* | Gamma-aminobutyric acid type A receptor gamma3 subunit |
|  |  |  |  | *GABRR1* | Gamma-aminobutyric acid type A receptor rho1 subunit |
|  |  |  |  | *GABRR3* | Gamma-aminobutyric acid type A receptor rho3 subunit (gene/pseudogene) |
|  |  |  |  | ***KCNJ6*** | Potassium voltage-gated channel subfamily J member 6 |
|  |  |  |  | *PLCL1* | Phospholipase C like 1 (inactive) |
|  |  |  |  | ***PRKCA*** | Protein kinase C alpha |
|  |  |  |  | ***PRKCB*** | Protein kinase C beta |
|  |  |  |  | *SLC12A5* | Solute carrier family 12 member 5 |
| Thyroid hormone signaling pathway | hsa04919 | 3.6194e-4 | 9.29753-3 | *AKT3* | AKT serine/threonine kinase 3 |
|  |  |  |  | ***ATP1B2*** | Atpase Na+/K+ transporting subunit beta 2 |
|  |  |  |  | ***ATP1B3*** | Atpase Na+/K+ transporting subunit beta 3 |
|  |  |  |  | *DIO3* | Iodothyronine deiodinase 3 |
|  |  |  |  | *ITGB3* | Integrin subunit beta 3 |
|  |  |  |  | *KAT2B* | Lysine acetyltransferase 2B |
|  |  |  |  | ***MAPK1*** | Mitogen-activated protein kinase 1 |
|  |  |  |  | *MED24* | Mediator complex subunit 24 |
|  |  |  |  | *NCOA2* | Nuclear receptor coactivator 2 |
|  |  |  |  | *PLCG1* | Phospholipase C gamma 1 |
|  |  |  |  | ***PRKCA*** | Protein kinase C alpha |
|  |  |  |  | ***PRKCB*** | Protein kinase C beta |
|  |  |  |  | *RXRA* | Retinoid X receptor alpha |
|  |  |  |  | *RXRG* | Retinoid X receptor gamma |
|  |  |  |  | *TBC1D4* | TBC1 domain family member 4 |
|  |  |  |  | *THRB* | Thyroid hormone receptor beta |
|  |  |  |  | *TP53* | Tumor protein p53 |

FDR, false discovery rate adjusted p-value. hsa, *Homo sapiens*. Genes that are present in different pathways are highlighted in bold.
